# The Story of the Insane from Year to Year

**Published:** 1890-09-20

**Authors:** 


### The Story of the Insane from Year to Year.

[Annual Reports, Pamphlets, &c., should be addressed to The Editor, The Lodge, Porchester Square, London, W.]

### CORK DISTRICT ASYLUM.

During the year 305 patients were admitted; 238 were discharged (164 having recovered), and 71 died. The year closed with 1,007 in the asylum. The percentage of recoveries reached 53·7, and the deaths were only 7 per cent. on the average number resident. A large dining hall 113 feet long by 89 wide has been opened, and the Privy Council has sanctioned a loan of £28,000 to build additions for 430 patients, a proceeding which can hardly be approved, in view of the 1,007 at present on the books, and that the erection of a separate asylum in another part of the county was advised by one of the lunacy inspectors. The committee do not print a report, but the inspector says, "With the general condition of the asylum, which I inquired into throughout its various departments, I am able to report in very favourable terms." The asylum is now superintended by Dr. Oscar Woods.

### DUNDEE ROYAL ASYLUM.

This asylum contains about 300 patients, and sixty or so are private patients. The recoveries stand at 33 33 per cent. on the admissions, and the deaths at 10·51 per cent. on the average number resident. It should, however, be stated that the average percentage of recoveries since the opening of the asylum has been considerably higher, and the percentage of deaths much lower than those for the year 1889. When speaking of criminal lunatics, Dr. Rorie says that Scotland should have "a criminal asylum equipped with all the modern appliances for the treatment as well as the care of the insane prisoners, similar to the asylum which has been already provided for England, at Broadmoor, and in which not only insane prisoners, but also those exceptionally dangerous cases which from time to time appear in ordinary asylums could be treated and specially cared for." This is exactly the point, and it is with reference to the latter that Broadmoor is useless to English medical superintendents. With a staff of one attendant to twelve patients it is well nigh impossible to watch homicidal lunatics, but no matter how dangerous a patient may be he cannot be got rid of until he has actually committed murder. There were five deaths from phthisis, two from pneumonia, and one from bronchitis. Dr. Rorie has had a fair share of changes in his staff. A new assistant medical officer, a new housekeeper, and a new head attendant have been appointed during the year. The Commissioners of Lunacy say, "the wards were found in excellent order and very clean, and they presented a bright and cheerful aspect."

### WONFORD HOSPITAL FOR THE INSANE, EXETER.

The report for 1889 shows much vigour in the management, both committee and medical superintendent being clearly determined to push the asylum into the foremost rank of kindred institutions. More land has been acquired; villa residences for patients are to be put up; a recreation and dining-room are to be erected; the laundry is to be improved, and the heating arrangements of the whole building overhauled. Dr. Deas is known as one of the ablest superintendents in England, and his report is as full of information as it is concise. The recoveries were 36 per cent. on the admissions, and the death rate only 4·2 per cent. on the average number resident. Influenza paid the asylum a visit and, curiously enough, confined its attention solely to the staff. The charitable object of the hospital is not forgotten, and we learn that 66 of the patients paid less than the actual cost, which number out of 117 must be considered satisfactory. We can endorse Dr. Deas' remarks on the much ventilated question of restraint. It should be used whenever there is good reason to believe that it is best for the patient. "I used it," says Dr. Deas, "both for the sake of the additional protection thus afforded to the patients against themselves, and also, I feel bound to say, to give some relief to the great, almost intolerable, strain and responsibility devolving on those having the care of such cases." "A physician, having the responsible care of the insane, ought to be as free to employ 'mechanical restraint,' when he deems it the best treatment in a particular case, as he is to use powerful drugs, or any other recognised mode of treatment." Exactly.

### ENNIS DISTRICT ASYLUM.

Like most of the Irish asylums, this is a small institution, containing space for 360 patients, and at the end of the year there were in it 340. During the year 79 cases were admitted, 50 were discharged, and 18 died. These numbers show that 36·7 per cent. recovered and 4·9 per cent. died, the latter being extremely low. No serious accident occurred during the year, and Dr. Gelston pays a tribute to his staff by saying that this immunity "reflects credit on the attendants for the vigilance with which they have discharged their respective duties." We could wish that such recognitions of the attendants were a little commoner than they are. The average cost per head was £20 6s. 1d. The inspector says that he found the asylum "in every respect worthy of commendation and most creditable to all connected with its control and management."

### OXFORD COUNTY ASYLUM.

Dr. Sankey's report is a short one, taking up barely four pages of rather large type. We learn from it that 523 patients were in residence at the close of the year; 149 were admitted, 44 were discharged recovered, and 61 died. Put into percentages, the recoveries stand at 31, and the deaths at 10. Only two patients were secluded, and the duration of the seclusion was only one hour and a quarter. This would seem very creditable. We also notice that *post-mortem* examinations were made in every case of death with one exception. The influenza visited the asylum, and 78 were affected, the staff being affected to a much greater extent, relatively, than the patients, a peculiarity which has been noted in more than one asylum. Dr. Sankey once more insists that "early treatment in an asylum means early recovery," and we hope he will be listened to. He would "make it a misdemeanour on the part of the relieving officer or the relations who did not take prompt action to get an insane person removed from their present surroundings." This might be a strong measure, but nothing short of it will be efficient. The committee state that as "the accounts of the asylum being now made subject to the audit of the Government auditor," they have made arrangements that the duties of their own salaried auditor should cease. The visitors must not forget that they have still to examine the asylum accounts, and it does not seem that they have, nor indeed ever had, the power to delegate this duty. The Commissioners in Lunacy say they "were well satisfied with the cleanliness of the wards and dormitories, though in several places the flooring is so much worn as to require renewal." They very properly advise that amusing books should be provided, and they think that too many of the volumes in use are "of a semi-religious character," a remark which has probably a wider range of application than the Oxford Asylum. The entertainments seem very few, but there is no "special room for social gatherings." It may be a small matter, but we think the Medical Superintendent's report should always follow that of the Committee of Visitors.

